# Telehealth delivery of adapted CBT-I for insomnia in chronic pain patients: a single arm feasibility study

**DOI:** 10.3389/fpsyg.2023.1266368

**Published:** 2024-01-11

**Authors:** Zoe Zambelli, Elizabeth J. Halstead, Antonio R. Fidalgo, Stephen Mangar, Dagmara Dimitriou

**Affiliations:** ^1^Sleep Education and Research Laboratory, Psychology and Human Development, UCL-Institute of Education, London, United Kingdom; ^2^School of Psychology, University of East London, London, United Kingdom; ^3^Department of Clinical Oncology, Charing Cross Hospital, Imperial College Healthcare NHS Trust, London, United Kingdom

**Keywords:** sleep, insomnia, feasibility and acceptability, CBT-I (cognitive behavioral treatment for insomnia), chronic pain

## Abstract

**Objectives:**

A large proportion of individuals with chronic pain experience insomnia-related symptoms which can be persistent in nature, and negatively impact one’s quality of life. This single arm trial aimed to investigate the feasibility and preliminary efficacy of CBT-I, adapted for people with chronic musculoskeletal pain, delivered via telehealth.

**Methods:**

We conducted a single arm feasibility trial in which 10 adult women (M age = 50.76 years, SD = 8.03 years) with self-reported insomnia and a diagnosed chronic musculoskeletal chronic pain received six CBT-I individual treatment sessions over 6–10 weeks. Treatment was delivered via telehealth. Participants completed weekly sleep diaries, and self-reported measures of insomnia, pain, anxiety and depression pre-treatment, post-treatment, and one-month follow-up.

**Results:**

The trial yielded, high levels of compliance with intervention protocols, and affirmative feedback on satisfaction which demonstrated feasibility. The enrolment rate into the study was 37% (27 participants screened, 10 participants enrolled). The intervention was associated with statistically and clinically meaningful improvements in self-reported insomnia severity. There were statistically significant improvements in sleep efficiency, wake after sleep onset, sleep onset latency, anxiety and depression.

**Conclusion:**

Adapted CBT-I delivered via telehealth may be a feasible, acceptable, and efficacious therapeutic approach for individuals with co-existent sleep and chronic pain. Future trials should adopt a randomized design against usual care.

## Introduction

Cognitive behavioral therapy for insomnia (CBT-I) is the recommended first-line treatment for chronic insomnia disorder by the British Sleep Society and the European Sleep Research Society, due to its demonstrated efficacy, effectiveness, low-risk profile, and improved long-term outcomes relative to pharmacotherapy (Lipman, 2017; O’Brien and Boland, 2020). The gold standard model of CBT-I typically consists of four to eight sessions one-to-one with a trained practitioner and has been shown to ameliorate sleep outcomes irrespective of medical and psychiatric comorbidities, age, and medication status (Soong et al., 2021).

There is a well-established bidirectional relationship between insomnia (characterized by difficulty falling or staying asleep, or unintentional early morning awakenings) and chronic pain (characterized by pain persistent beyond normal healing time for a duration of 3 months or more), with evidence suggesting that poor sleep can exacerbate pain symptoms, and in turn, chronic pain can negatively impact sleep quality and continuity (Woo and Ratnayake, 2020). Non-malignant pain conditions are prevalent and affect up to one third of adults in the UK, with a high percentage (up to 88%) of these individuals experiencing insomnia related symptoms (Smith et al., 2000; Fayaz et al., 2016).

CBT which includes components specifically targeted for insomnia have been shown to be effective in improving several parameters of sleep such as sleep efficiency and subjective sleep quality in numerous systematic reviews reporting both statistically and clinically meaningful results (Mitchell et al., 2012; Trauer et al., 2015). Efforts have been made to address sleep disturbances in clinical populations formerly termed “secondary insomnia,” and with this, a surge of RCT and pilot studies have been conducted to assess the efficacy of CBT-I for both sleep and pain outcomes (Lichstein, 2006; Selvanathan et al., 2020). These results have been replicated among other clinical populations with comorbid insomnia such as cancer and psychiatric populations (Garland et al., 2014; Taylor and Pruiksma, 2014).

### Telehealth and CBT-I

The rapid spread of telehealth interventions, accelerated by the COVID-19 pandemic, has presented healthcare providers and researchers with a unique opportunity to save on delivery costs of health-related interventions, while potentially increasing reach and accessibility to marginalized groups of patients (Gajarawala and Pelkowski, 2021).

Several studies have now had the opportunity to evaluate the acceptability of telehealth in a range of clinical disciplines, across adult and pediatric populations, and in real-world settings (Tse et al., 2021; Cuthbert et al., 2022). For example, several models of psychological therapy have been delivered and evaluated utilizing telehealth and found to be acceptable, and importantly, addressing a health disparity gap by overcoming geographical barriers and supporting vulnerable remote populations (Cuthbert et al., 2022).

Some work has been conducted to compare efficacy of telehealth delivery vs. traditional face-to-face CBT-I (Gehrman et al., 2021). The effectiveness of CBT-I and adapted CBT-I on sleep outcomes among people with non-malignant chronic pain, in addition to the high levels of acceptability of telehealth interventions in behavioral medicine provide a strong rationale for developing an adapted CBT-I intervention, delivered via telehealth. Furthermore, the NG-193 guidelines (NICE, 2021) have made a key research recommendation to increase the evidence base for CBT-I in the treatment of chronic pain (Zambelli et al., 2022).

## Aims of the current study

The primary aim of this study was to assess the feasibility of an adapted CBT-I intervention for individuals with chronic pain by examining:

1. Recruitment (i.e., willingness to participate in the intervention)
2. Compliance and attrition rates throughout the study period
3. Participant satisfaction with the intervention

The second aim was to assess the extended benefits (one-month post-treatment) of the intervention to explore potential efficacy. Intervention benefits were assessed using:

1. Subjective sleep outcomes based on sleep diary data and the Insomnia Severity Index:
2. Pain severity and interference outcomes, depression, and anxiety (quality of life) from the Brief Pain Inventory and the Hospital Anxiety and Depression Scale.

## Methods

### Design

Given the predominant focus on feasibility and acceptability, we chose to use a pre-post single-arm trial design with three measurement points (baseline, immediate post-treatment, and one-month follow-up).

Adult women with musculoskeletal chronic pain (MSK; pain typically affecting bones, joints, ligaments, and tendons) were recruited over a two-month period June–July 2022 from an opt-in mailing list created during a previous UK-wide survey (Zambelli et al., 2022).

All participants provided voluntary informed consent prior to the initiation of the study. The study was approved by UCL’s Institute of Education Ethics Review Committee, reference: Z6364106/2022/02/136. This feasibility study was pre-registered via the Open Science Framework: https://osf.io/q5y9x.

Participants were included if they provided consent, were willing to comply with all study procedures, aged between 25 and 65 (working aged adults), were able to communicate in English, resided in the UK, self-reported chronic pain for more than 6 months, received a clinical diagnosis of non-malignant chronic pain affecting their: lower back, knee, hip, shoulder, neck, had a stable pain medication regime, had access to a computer or tablet with stable internet connection as well as a web camera. Finally, participants were included if had an insomnia score of above 8, indicated through screening procedures which were scored by the research team.

Exclusion criteria were: if participant was self-reporting medication or dosage change in the previous 1 month, had the presence of clinically assessed self-injury, current suicidal intent, self-reported borderline personality disorder or any form of schizophrenia, epilepsy, bipolar disorder or dementia spectrum disorders, additional intellectual disability or cognitive and/or genetic impairment that would prevent them from understanding the research protocol, self-reported history or current substance abuse, self-reported clinical diagnosis of unstable restless leg syndrome or period limb movement disorder, currently receiving any form of CBT, overnight shift work in the past 3 months, or pregnant / breastfeeding.

**Figure 1:**
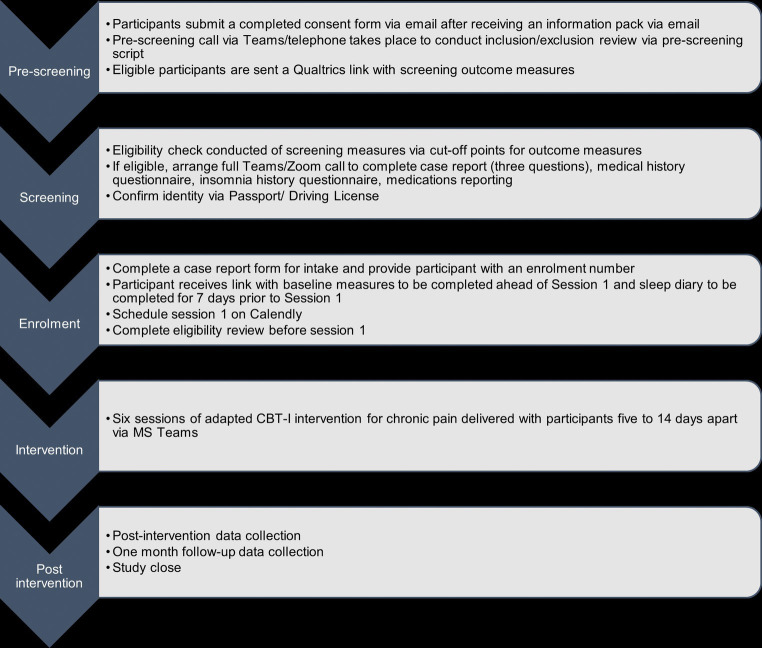
Study procedure flowchart.

Recruitment took place between June and July 2022, and data collection was completed by November 2022. Figure 1 displays the study procedure flowchart.

### Intervention

The intervention consisted of six individual treatment sessions, including sleep hygiene, stimulus control (adapted for chronic pain), sleep restriction (adapted for chronic pain), as well as additional components targeted specifically to MSK pain, such as relaxation techniques, psychoeducation, and pain management in the context of insomnia. The traditional guidelines for sleep restriction and stimulus control were adapted to make consideration for the impact of chronic pain on an individual’s mobility and bedtime routine (Soong et al., 2021).

The adapted CBT-I intervention protocol was manualized and followed a strict treatment protocol, aiming to keep fidelity to the original aims of CBT-I: reducing beliefs and behaviors that perpetuate insomnia and improving overall sleep quality. The adapted intervention had an average duration of 50 min per session, bar the first session of 90 min. Supplementary File S1 provides an outline of the intervention content by session.

### Delivery and quality assurance

The intervention sessions were delivered via Microsoft Teams. Participants were encouraged to keep their webcam on during each session with the research therapist. If the participant did not wish to have the webcam on, this would not exclude or disadvantage the participant. However, having the webcam on was viewed as closer to traditional face-to-face sessions and provided the research therapist with possible indications of anxiety signs. Participants did not have to register for an account to join the sessions. All sessions were manualized and delivered by ZZ, who received CBT-I practitioner training prior to commencement. Supervision was provided in a case conference format by LH, a BACP-accredited psychologist and trained CBT-I practitioner.

### Participants

Participants were working-aged women aged between 38 and 65 (*M* = 50.76 years, *SD* = 8.03 years). Participants had received diagnosis of MSK pain in secondary care. Most participants reported their pain duration as more than 10 years (*n* = 6) and were taking medication as part of their pain management regime (*n* = 7). Participant characteristics are displayed in Table 1.

**Table 1:** Participant characteristics.

Variable	*n* (%)
Age^a^	50.67 (8.03)
Sex	
Female	9 (100%)
Ethnicity	
White—any white background	9 (100%)
Education attainment	
Higher, secondary, or further education Postgraduate degree	3 (33%) 6 (67%)
Employment status	
Employed (full-time) Employed (part-time) Self-employed Unemployed on disability allowance	1 (11%) 3 (33%) 3 (33%) 2 (22%)
MSK pain	
Chronic Widespread Pain Hip osteoarthritis Lower back pain	7 (78%) 1 (11%) 1 (11%)
Diagnosis	
Speciality doctor	9 (100%)
Chronic pain duration	
>3 to <5 years >5 to <10 years Over 10 years	1 (11%) 2 (22%) 6 (67%)
Pain medication	
Yes Of which opiate analgesics No	7 (78%) 6 (86%) 2 (22%)

^a^Continuous variable for the age of participants displays the M(SD).

### Measures

#### Insomnia severity index (screening, baseline, post-intervention, follow-up)

The ISI (Bastien et al., 2001) is a seven-item scale that provides a subjective report of insomnia and is validated to evaluate insomnia severity in reference to the diagnostic criteria for primary insomnia from the DSM-IV. Each item is rated on a 4-point Likert scale from 0 to 4 with higher scores indicating more acute symptoms of insomnia. A total score of 0–7 indicates “No clinically significant insomnia,” 8–14 “Subthreshold insomnia,” 15–21 “Clinical insomnia (moderate severity)” and 22–28 “Clinical insomnia (severe).” The ISI has shown good internal consistency with Cronbach’s alpha of 0.90 (Bastien et al., 2004).

#### PHQ-9 (screening measure)

The PHQ-9 (Kroenke et al., 2001) is a 9-item depression screening tool scoring from 0 to 27, with higher scores indicating more severe depression. Validated for spinal injury, migraine, and back pain cohorts, it is used in general and mental health settings with Cronbach’s alpha of at least 0.70 (Lotrakul et al., 2008; Bombardier et al., 2012; Seo and Park, 2015; Suni et al., 2021).

#### Sleep apnoea risk (screening measure)

The STOP-BANG questionnaire (Chung et al., 2008) is an 8-point screening tool for obstructive sleep apnoea risk, with scores >3 indicating high risk. Validated for general and clinical populations, it considers factors like BMI, age, neck size, and gender (Nagappa et al., 2015). The STOP-BANG has shown good internal consistency with Cronbach’s alpha of 0.8 (Delgado-Vargas et al., 2021).

#### CORE-OM (screening measure)

The CORE-OM (Barkham et al., 1998) is a 34-item self-report measure covering four domains: subjective well-being, problems/symptoms, life functioning, and risk to self and others. It employs a 5-point scale, demonstrating good psychometric properties with Cronbach’s alpha between 0.75 and 0.95 (Evans et al., 2002).

#### Brief pain inventory (baseline, post-intervention, follow-up)

The BPI is measure for clinical pain (Cleeland and Ryan, 1991). The BPI includes two subscales which rate the severity of pain and pain interference. Subscales range from 0 to 10 with higher scores indicating higher levels of pain severity and pain interference. These scales have good internal consistency with Cronbach’s alpha of 0.85 and 0.88 for the severity and interference scales, respectively (Tan et al., 2004).

#### Epworth sleepiness scale (baseline, at each session, post-intervention, follow-up)

The ESS (Johns, 1990) evaluates daytime sleepiness and fatigue. Items are rated on a 3-point Likert scale from 0 (would never doze) to 3 (high chance of dozing). The higher the score, the higher the respondents’ “daytime sleepiness.” The scale is used both for research purposes and clinically. The ESS has shown good reliability with Cronbach’s alpha of 0.74–0.88 (Johns, 1994).

#### Hospital anxiety and depression scale (baseline, post-intervention, follow-up)

The HADS is a 14-item validated measure (Zigmond and Snaith, 1983). It comprises of two subscales for anxiety (HADS–A) and depression (HADS–D). Items are rated on a four-point Likert scale (e.g., 0 = not at all to 3 = most of time). Scores above eight suggest clinical anxiety and depression symptoms. The HADS measure has been validated for use in non-malignant chronic pain populations (Castro et al., 2006; Turk et al., 2015). The HADS has shown good internal consistency with Cronbach’s alpha of 0.84 for HADS-A and 0.82 for HADS-D (Kemani et al., 2016).

#### Sleep diary (baseline, post-intervention, follow-up)

Participants completed electronic daytime and night-time sleep diaries daily during the intervention period and during follow-up. The daytime diary included 17 questions about factors affecting participants’ day, including exercise and alcohol/caffeine consumption. Participants rated their daily fatigue, stress, alertness, concentration, mood, and pain levels. The night-time diary had 10 questions about overnight factors, such as sleep quality, number of awakenings, and time taken to fall asleep. Participants rated their sleep quality and morning refreshment. Sleep diaries were completed electronically and provided in Excel format.

Sleep efficiency was chosen as the primary outcome of the sleep diary, as it is a summary measure that considers multiple sleep diary parameters. It was calculated by dividing total sleep by time in bed and multiplying by 100. Other sleep parameters included in the analysis were sleep onset latency (SOL), wake after sleep onset (WASO), and total sleep time (TST). These were calculated as means across 7 days and nights of data.

#### Participant satisfaction questionnaire (post intervention)

In order to assess participants’ experience of the intervention and its format, an experience and acceptability questionnaire consisting of 13 items was designed. The questionnaire was included in the post-intervention survey.

The themes explored overall satisfaction with the intervention, the likelihood to recommend the intervention to friends and family with insomnia and chronic pain, potential improvements to the intervention, and the perceived impact of the intervention on participants.

### Analyses

Feasibility was assessed using frequency analysis which examined the study recruitment rate calculated by dividing the number of participants who completed the study, by the number of participants enrolled. Compliance rates were examined by reporting the completion rates of the study procedure, follow-up completion rate, and rescheduling rates. These rates were expressed as percentages or whole numbers due to the small sample size.

To assess within-group treatment effects on primary outcomes of sleep, and subsequently, secondary outcomes related to pain severity, pain interference, depression, and anxiety a repeated measures ANOVA model was used to generate parameter estimates to determine the significance of changes across time points at baseline, post-intervention, and one-month follow-up for each outcome of interest. The data were found to have met assumptions for normality and sphericity before carrying out repeated measures analyses utilizing the Shapiro–Wilk test.

Effect size using Partial Eta Squared was reported (small *d* = 0.2, medium *d* = 0.5, and large *d* = 0.8; Cohen, 1988). Data were analyzed on IBM SPSS v. 28.

## Results

### Feasibility: study recruitment

**Figure 2:**
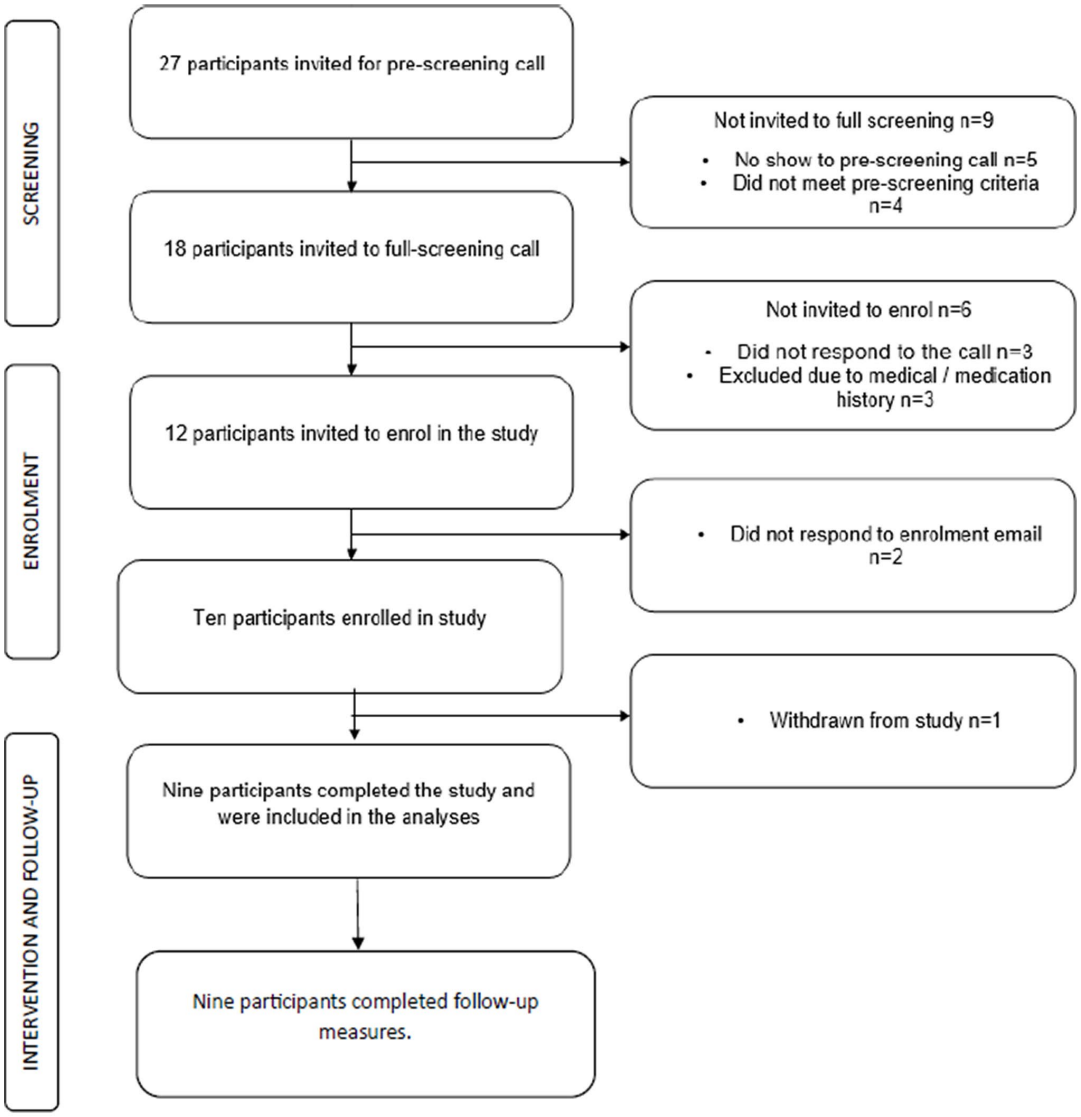
Consort diagram.

There were 27 participants who came forward and were pre-screened for their eligibility. Of these, 10 participants (37% recruitment/enrolment rate) were enrolled in the study after the pre-screening and full-screening procedure as per criteria outlined here. Five participants did not show up for pre-screening, four were deemed ineligible during pre-screening, and three did not respond to scheduling requests for the full-screening call. Of the 15 participants who attended the full-screening call, three were excluded due to medical history disclosures, and two did not respond to follow-up emails. One participant was later withdrawn due to noncompliance and referred to a sleep clinic for a suspected sleep disorder unrelated to insomnia (see Figure 2 consort flowchart).

### Feasibility: compliance

One participant was excluded from the study after three sessions due to suspected paradoxical insomnia which caused an inability to comply with the study procedures as per exclusion criteria. This participant completed three further adapted sessions with no sleep restriction component, retaining only information about relaxation/ sleep hygiene techniques.

Of the remaining nine participants, 100% completed the six intervention sessions, and weekly sleep diaries, and completed the follow-up measures and post intervention and 1 month after session six.

Four sessions out of 36 intervention sessions (11%) among the nine final participants were rescheduled due to illness (*n* = 1), annual leave (*n* = 2) and scheduling conflicts (*n* = 1).

### Feasibility: participant satisfaction with the intervention

All nine participants who completed the study were able to complete the participant satisfaction survey, part of the post-intervention online questionnaire. All nine participants (100%) were “very satisfied” with the overall intervention, on a scale of 0 = very unsatisfied, to 4 = very satisfied. Furthermore, all nine participants (100%) reported they would be “very likely” to recommend the adapted CBT-I intervention to an adult with chronic pain and concomitant insomnia on a scale of 0 = not likely at all, to 4 = very likely.

Participants were asked how far they agreed with seven statements on a scale of 0 = do not agree at all, to 4 = completely agree. All participants agreed that the therapist was able to communicate in a clear way throughout the intervention, trusted the therapist throughout the intervention, the therapist was knowledgeable about sleep and chronic pain conditions, and importantly, participants felt more empowered to manage their sleep problems in the present and the future.

With regards to whether the intervention met the participants’ expectations, seven participants completely agreed with this statement, one participant “mostly agreed,” and one participant “neither agreed nor disagreed.” Eight participants completely agreed that the intervention sessions had been tailored to participants’ individual sleep goals, and one participant “mostly agreed.” Finally, eight participants completely agreed that they themselves felt more knowledgeable about sleep problems and how they interact with chronic pain.

### Treatment effects on primary sleep outcomes

**Figure 3:**
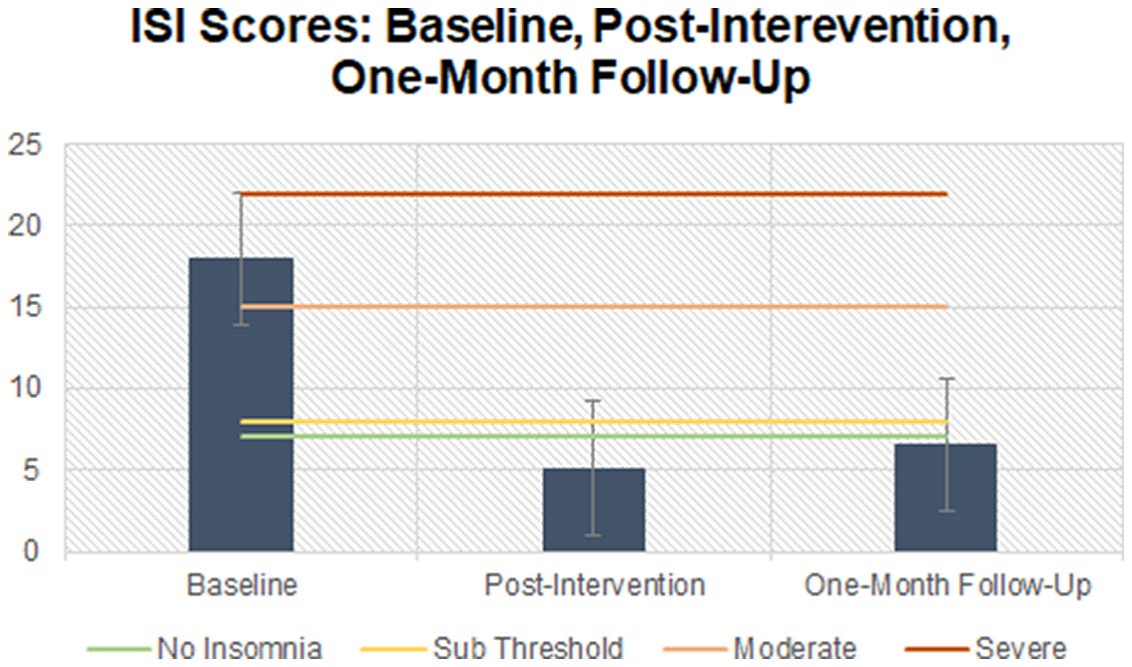
ISI scores between baseline, post-intervention, and follow-up.

**Figure 4:**
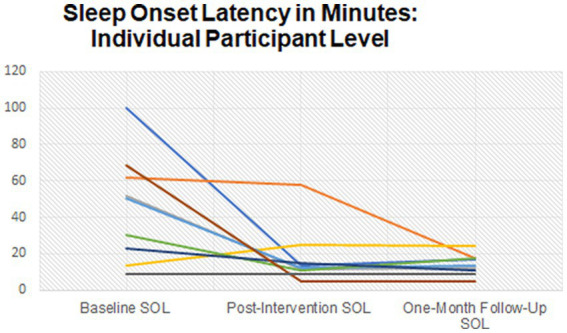
Sleep onset latency between baseline, post-intervention, and follow-up.

**Figure 5:**
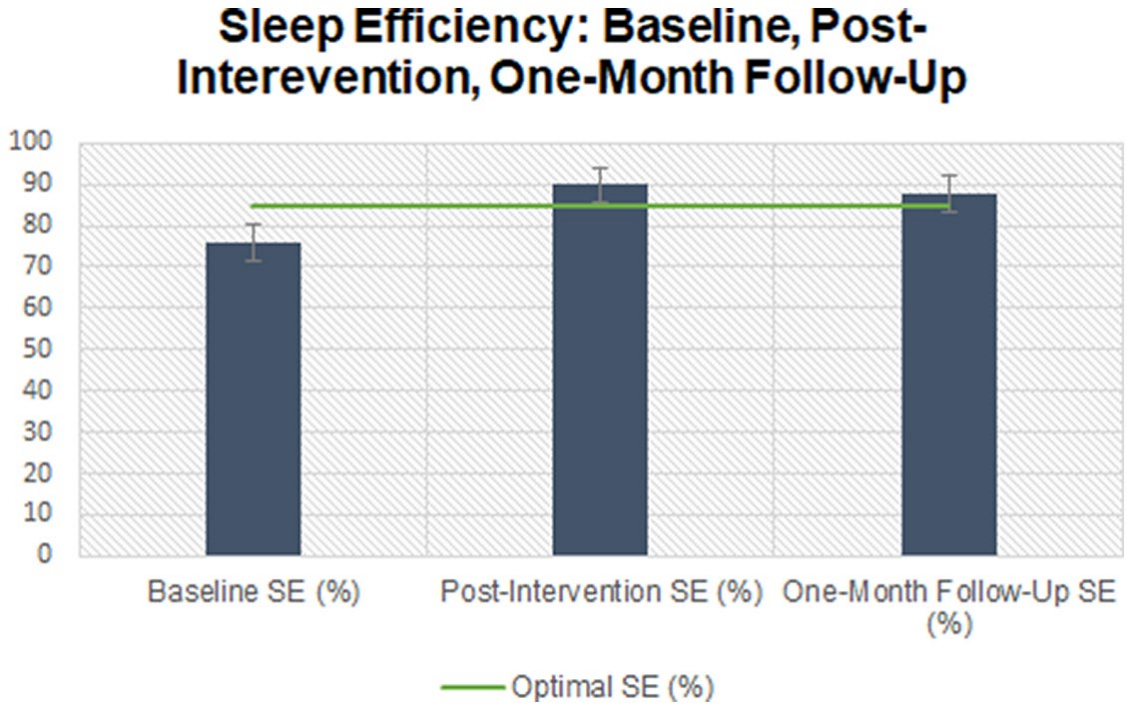
Sleep efficiency between baseline, post-intervention, and follow-up.

The majority (*n* = 7) of participants began treatment with a combination of onset and maintenance insomnia. One participant began treatment with onset insomnia only, and one participant began treatment with early morning awakenings. Supplementary File S2 displays the sleep profiles for each of the nine participants who completed the study and attended all six sessions of CBT-I. Figure 3 demonstrates the mean ISI scores between baseline and follow-up which began above threshold for Moderate insomnia and fell to within “no insomnia” range at follow-up. Figure 4 demonstrates changes in individual SOL between baseline and follow-up. Figure 5 shows the increases in mean SE between baseline and follow-up.

Repeated measures ANOVAs were conducted to determine whether there were statistically significant differences in insomnia severity, SOL, WASO, TST, and sleep efficiency over the course of the intervention. Table 2 displays the descriptive statistics across the primary sleep outcomes of interest at baseline, post-intervention, and one-month follow-up.

**Table 2:** Baseline, post-intervention, and one-month follow-up outcomes related to sleep.

Outcome measure	Baseline M (*SD*)	Post-intervention M (*SD*)	One-month follow-up M (*SD*)
Insomnia severity index score	18 (5.57)	5.58 (1.72)	6.56 (4.12)
Sleep onset latency in minutes	45.36 (29.54)	17.68 (16.03)	14.13 (5.74)
Wake after sleep onset in minutes	45.09 (36.90)	17.02 (13.83)	21.86 (18.59)
Total sleep time in minutes	384.79 (89.81)	417.71 (42.67)	428.27 (65.47)
Sleep efficiency (%)	75 (15.30)	89.81 (7.30)	87.81 (8.31)

The first model yielded statistically significant changes in insomnia severity as measured through the ISI, *F*(2, 16) = 45.13, *p* < 0.001, partial *η*^2^ = 0.849, with insomnia severity scores decreasing from 18 ± 5.57 at baseline to 5.58 ± 1.72 at post-intervention and to 6.56 ± 5.74 at one-month follow-up. *Post hoc* analysis with a Bonferroni adjustment revealed that insomnia severity was statistically significantly decreased from baseline to post-intervention [12.89 (95% CI, 7.08–17.98), *p* < 0.001], and from baseline to one-month follow-up [11.44 (95% CI, 6.84–16.05)*, p* < 0.001], but not from post-intervention to one-month follow-up [−1.44 (95% CI, −5.07 to 2.18), *p* = 0.793].

The second model yielded statistically significant changes in sleep onset latency, measured through the sleep diary contents, over time, *F*(2, 16) = 7.29, *p* = 0.006, partial *η*^2^ = 0.477, with sleep onset latency decreasing from an average of 45.36 ± 29.54 min at baseline to 17.68 ± 16.08 min at post-intervention and to 14.13 ± 5.74 min at a one-month follow-up. *Post hoc* analysis with a Bonferroni adjustment revealed that sleep onset latency was statistically significantly decreased from baseline to one-month follow-up [31.23 (95% CI, 6.11–61.85) minutes, *p* = 0.046], but not from baseline to post-intervention [26.68 (95% CI, −4.76 to 60.11) minutes, *p* = 0.099], nor from post-intervention to one-month follow-up [3.56 (95% CI, −10.54 to 17.65) minutes, *p* = 1.00].

The third model yielded statistically significant changes in wake after sleep onset, measured through the sleep diary contents, over time, *F*(2, 16) = 3.64, *p* = 0.50, partial *η^2^* = 0.313, with wake after sleep onset decreasing from an average of 45.09 ± 36.90 min at baseline to 17.02 ± 13.83 min at post-intervention and to 21.86 ± 18.59 min at one-month follow-up. *Post hoc* analysis with a Bonferroni adjustment revealed that sleep onset latency was not statistically significantly decreased from baseline to post-intervention [28.07 (95% CI, −10.12 to 66.27) minutes, *p* = 0.17], nor from baseline to one-month follow-up [23.23 (95% CI, −16.81 to 63.28) minutes, *p* = 0.36], nor from post-intervention to one-month follow-up [−4.84 (95% CI, −22.63 to 12.95) minutes, *p* = 1.00].

The fourth model yielded non-statistically significant changes in total sleep time, measured through the sleep diary contents, over time*, F*(2, 16) = 1.79, *p* = 0.199. *Post hoc* analyses were not consulted.

Finally, the last model yielded statistically significant changes in sleep efficiency, measured through the sleep diary contents, over time, *F*(2, 16) = 5.41, *p* = 0.02, partial *η*^2^ = 0.404, with sleep efficiency increasing from an average of 75 ± 15.30 per cent at baseline to 89.81 ± 7.30 per cent at post-intervention and to 87.81 ± 8.31 per cent at one-month follow-up. *Post hoc* analysis with a Bonferroni adjustment revealed that sleep efficiency was not statistically significantly decreased from baseline to post-intervention [−13.88 (95% CI, −30.97 to 3.21) per cent, *p* = 0.12], nor from baseline to 1-month follow-up [−11.88 (95% CI, −26.79 to 3.04) per cent, *p* = 0.13], nor from post-intervention to one-month follow-up [2.00 (95% CI, −5.26 to 9.27) per cent, *p* = 1.00].

### Treatment effects on pain and quality of life outcomes

Repeated measures ANOVAs were conducted to determine whether there were statistically significant differences in secondary clinical outcomes of interest: pain severity, pain interference, anxiety, and depression scores over the course of the intervention and follow-up period. The data were assessed for normality utilizing the Shapiro–Wilk test and all variables were found to be normally distributed (*p* > 0.05). Table 3 displays the descriptive statistics for all secondary quality of life outcomes of interest at baseline, post-intervention, and one-month follow-up.

**Table 3:** Baseline, post-intervention, and one-month follow-up outcomes related to pain and quality of life.

Outcome measure	Baseline M (*SD*)	Post-intervention M (*SD*)	One-month follow-up M (*SD*)
Pain severity	5.50 (1.88)	5.58 (1.72)	5.11 (2.60)
Pain interference	5.84 (2.07)	5.48 (1.93)	5.19 (2.59)
Anxiety	10.22 (5.43)	6.22 (3.67)	7.00 (4.30)
Depression	10.00 (3.16)	5.56 (3.64)	6.56 (4.10)

The first model yielded non-statistically significant changes in pain severity, measured through the brief pain inventory, over time*, F*(2, 16) = 0.38, *p* = 0.689. *Post hoc* analyses were not consulted due to the non-significant result.

The second model yielded non-statistically significant changes in pain interference, measured through the brief pain inventory, over time, *F*(2, 16) = 0.64, *p* = 0.541. *Post hoc* analyses were not consulted due to the non-significant result.

The third model yielded statistically significant changes in anxiety, measured through the hospital anxiety and depression scale (HADS), over time, *F*(2, 16) = 4.87, *p* = 0.022, partial *η*^2^ = 0.378, with anxiety scores decreasing from 10.22 ± 5.43 at baseline to 6.22 ± 3.67 at post-intervention and to 7.00 ± 4.30 at one-month follow-up. *Post hoc* analysis with a Bonferroni adjustment revealed that anxiety scores were not statistically significantly decreased from baseline to post-intervention [4.00 (95% CI, −1.75 to 8.18), *p* = 0.061], nor from baseline to one-month follow-up [3.22 (95% CI, −1.85 to 7.18), *p* = 0.26], nor from post-intervention to one-month follow-up [−7.88 (95% CI, −3.47 to 1.91)*, p* = 1.00].

The fourth and final model yielded statistically significant changes in depression, measured through the hospital anxiety and depression scale, over time, *F*(2, 16) = 7.87, *p* = 0.004, partial *η*^2^ = 0.496, with depression scores decreasing from 10.00 ± 3.16 at baseline to 5.56 ± 3.64at post-intervention and to 6.56 ± 4.10 at one-month follow-up. *Post hoc* analysis with a Bonferroni adjustment revealed that depression scores were statistically significantly decreased from baseline to post-intervention [4.44 (95% CI, 0.61–8.28), *p* = 0.04], but not from baseline to one-month follow-up [3.44 (95% CI, −0.29 to 7.18), *p* = 0.71], nor from post-intervention to one-month follow-up [−1.00 (95% CI, −4.01 to 2.02), *p* = 1.00].

## Discussion

This study aimed to investigate the feasibility and preliminary efficacy of CBT-I, adapted for adults with chronic MSK pain. The study evaluated several domains which included recruitment outcomes, attrition rates, compliance, and participant satisfaction with the intervention. To assess the preliminary efficacy of the intervention in relation to sleep and quality of life outcomes, questionnaires and sleep diary data were analyzed.

The findings from this study demonstrate the feasibility and acceptability of a six-session adapted CBT-I intervention delivered via telehealth to improve sleep outcomes among adults with chronic MSK pain. Firstly, our recruitment-to-enrolment rate of 37% is similar to other recent studies which have conducted feasibility trials for CBT-I (Clarke et al., 2015; Law et al., 2018).

Participants demonstrated high levels of compliance with the intervention protocol, with nine out of ten enrolled participants able to fully adhere to treatment prescription, attendance, completion of sleep diaries, and follow-up measures. This high level of compliance is congruent with the positive feedback from the participant satisfaction survey, with all participants reporting the highest levels of overall satisfaction and likely to recommend the intervention to others. Participants reported a trusting relationship with the therapist and acknowledged the therapist’s expertise in the field of sleep and chronic pain.

Research has demonstrated the importance of therapeutic alliance, and its correlation with clinical outcomes in distinct therapeutic models and clinical populations (Leahy, 2008; Priebe et al., 2011; Pihlaja et al., 2018). Despite a small sample, the successful recruitment process, high levels of compliance with intervention protocols, and affirmative feedback on satisfaction indicate that this adapted model of CBT-I, delivered via telehealth, may be a feasible intervention for the target cohort.

Beyond feasibility, we aimed to examine the preliminary clinical efficacy of this adapted CBT-I intervention on sleep outcomes primarily, in addition to pain and mood levels. Across the sample, insomnia severity decreased significantly from baseline to post-intervention, and from baseline to follow-up. Not only were these changes statistically significant, but these were clinically meaningful, with a reduction of 12 and 11 points on the ISI, respectively (Wilson et al., 2016). Furthermore, examining individual-level progress, all nine participants reported sustained improvements in ISI scores between baseline and post-intervention, and follow-up, and eight participants fell below the ISI threshold for insomnia at post-intervention.

Additionally, sleep onset latency increased significantly across the sample between baseline and post-intervention, as did sleep efficiency. Night-time wakefulness decreased across the sample significantly throughout the study. An increase in time spent asleep during the night was observed from baseline (6.4 h) to post-intervention (7 h) and follow-up (7.14 h). Although these changes did not reach statistical significance, the trends suggest a positive effect and echo previous CBT-I results which yielded significant and positive changes in sleep outcomes (Pigeon et al., 2012; Tang et al., 2012; Smith et al., 2015; Lami et al., 2018). Above this, it is particularly encouraging to be able to report clinically meaningful results considering the sample size in this study.

Pain interference levels were not changed significantly throughout the study period, however, the trends demonstrated there were reductions in pain interference between baseline and post-intervention, and a further reduction at follow-up. In contrast to the trends observed in the sleep outcomes, pain outcomes continued to improve at follow-up, and it is plausible that if the follow-up period were longer, these may have yielded a statistically significant result, as has been reported previously (Law et al., 2018).

There is research which shows that insomnia, depression, and anxiety are likely to be interrelated in a similar way to sleep and pain and are mutual risk factors for the development of one another (Johnson et al., 2006; Benca and Peterson, 2008). It was therefore expected that our study sample reported mean scores of clinically mild anxiety and depression symptoms at baseline. Previous research has found that sleep quality moderated the relationship between pain interference and depression scores (Zambelli et al., 2021) and our sample’s scores for depression decreased significantly, and clinically meaningfully between baseline and post-intervention. Scores for anxiety decreased significantly throughout the study too. These results replicate previous CBT-I outcomes which measured mood and reported a positive impact on depression and anxiety outcomes (Taylor and Pruiksma, 2014).

Our data may also be used to validate the NICE recommendations (NG-193) regarding the use of psychologically informed practice as an effective form of treatment for coexisting insomnia in this population (Zambelli et al., 2022).

### Limitations

This study adopted a single-arm design which makes it difficult to infer whether any observed improvements in sleep and mood outcomes were due to the intervention, other care provisions the individuals may have received beyond the study (private care, NHS), or simply the passing of time. Secondly, this study aimed to assess feasibility and acceptability as its primary aim, therefore a small target sample of 10 female participants with chronic MSK pain was sufficient, however, looking to the future, it would be beneficial to recruit a larger and perhaps more diverse chronic pain sample to enable sub-group analyses across different demographics, pain conditions, and pain complexity, as these factors have been shown to as independent risk factors for sleep disturbance and may impact the trajectory of a CBT-I treatment on sleep outcomes (Rollman and Lautenbacher, 2001; Smith et al., 2001). In order to validate the findings from this study further, a larger study adopting a randomized control design should be conducted.

Finally, the telehealth delivery may pose a limitation to individual who are digitally excluded from participation, e.g., without access to internet, or adequate internet-connected devices with which to participate in such interventions. However, telehealth may be used to serve individuals who are unable to participate in in-person/on-site treatment due to reasons such as mobility impairments, occupational or caring responsibilities, or personal preference.

### Strengths

Within NHS services, talking therapies for chronic pain and insomnia are typically offered as distinct interventions, necessitating separate referrals. Moreover, when sleep is addressed within a pain management program, evidence indicates that the sleep component is not personalized, consisting primarily of sleep hygiene recommendations, if at all (Cunningham et al., 2011; Burton and Shaw, 2015). Our adapted CBT-I intervention aimed to address sleep problems in the context of living and managing chronic MSK pain with preliminary evidence that it may ameliorate outcomes in sleep and mood which may lead to fewer healthcare interactions and address high-cost barriers to care. Furthermore, the telehealth delivery of the intervention may help to address other barriers known to prevent targeted care provision, such as a lack of trained practitioners in certain geographical areas and increased accessibility for individuals living remotely (Gajarawala and Pelkowski, 2021).

### Recommendations

The findings from this feasibility study can provide clinical and policy implications, therefore we have listed some recommendations for consideration:

1. Firstly, healthcare practitioners treating patients with chronic MSK pain should be prepared to screen for insomnia disorder routinely, using validated outcome measures and technology where possible. When a patient shares their concern regarding their sleep, referral for a behavioral sleep intervention which includes the principal components of CBT-I could be considered as an option. Patients open to behavioral treatments should be screened for any other sleep conditions to ensure it can be effective.
2. Second, the findings highlight that it is feasible to deliver an adapted CBT-I for female adults with concomitant chronic MSK pain and insomnia and that this form of treatment is highly acceptable. Healthcare practitioners treating chronic pain patients should receive training and support to screen and, where appropriate, treat and monitor sleep disturbance in this population.
3. Third, telehealth delivery of CBT-I may produce large effects on sleep, and mood, similar to effects produced in face-to-face interventions which aim to sleep and mood. Healthcare commissioners and providers should seek to embed behavioral sleep medicine in routine pain management clinics.
4. The results from this study validate the NG-193 recommendation for utilizing psychological therapies in this population, and contribute toward building a larger evidence-base for their key research recommendations which names CBT-I as a promising therapeutic model.

## Conclusion

The results from this feasibility study highlight the potential benefits that an adapted CBT-I intervention can produce positive sleep and mood outcomes in female adults with chronic MSK pain. There is an abundance of literature which demonstrates the high prevalence of sleep disturbances among this population, and the detrimental impact of poor sleep on overall quality of life.

It is hoped that this feasibility study may contribute toward wider knowledge and acceptance of behavioral sleep medicine in the pain community. Future research should aim to build on the current findings, utilizing robust research design, and working with practitioners and educators to embed this into practice.

## Data availability statement

The raw data supporting the conclusions of this article will be made available by the authors, without undue reservation.

## Ethics statement

The studies involving humans were approved by the IOE, University College London Ethics Committee. The studies were conducted in accordance with the local legislation and institutional requirements. The participants provided their written informed consent to participate in this study.

## Author contributions

ZZ: Conceptualization, Data curation, Formal analysis, Funding acquisition, Investigation, Methodology, Project administration, Resources, Software, Validation, Visualization, Writing – original draft, Writing – review & editing. EH: Conceptualization, Methodology, Supervision, Writing – review & editing. AF: Conceptualization, Supervision, Writing – review & editing. SM: Validation, Writing – review & editing. DD: Conceptualization, Supervision, Writing – review & editing.
