# Supplementary material for: Telehealth delivery of adapted CBT-I for insomnia in chronic pain patients: a single arm feasibility study
Source: Front Psychol. 2024 Jan 11;14:1266368. doi: 10.3389/fpsyg.2023.1266368 (PMC10808483; doi:10.3389/fpsyg.2023.1266368)
Supplement: Supplementary file 1 [file Data_Sheet_1.PDF]

Supplementary File A.1 Intervention Outline

Session	Content
Session 1 (90 minutes)	• Introduction<br>• Goal setting and participant expectations<br>• Basic sleep physiology and circadian rhythm<br>• Spielman's Model of Insomnia<br>• Sleep and pain interaction<br>• Pain management strategies and their potential impact on sleep: medication, exercise, self-management<br>• Review of the sleep diary<br>• Making small changes – next steps
Session 2 (50 minutes)	• Goal setting<br>• Review of the sleep diary<br>• Review of 'making small changes'<br>• Education on healthy sleep practices commonly termed sleep hygiene. This part of the session refers to an infographic based on previous research, implications for pain management are discussed here<br>• Introduction to sleep restriction (referred to by participants as 'sleep scheduling'. Sleep restriction involves restricting the amount of time a participant spends in bed with subsequent steps to gradually increase the time spent in bed over the proceeding weeks. The initial bedtime is usually the average nightly total sleep time over the previous week (as determined by the 7-day sleep diary). However, the time allowed in bed will never be less than five hours as less than this can have a serious impact on the ability to function properly<br>• Calculating new sleep schedule with the participant and agreement to adhere to their new schedule including discussion and identification of potential barriers and facilitators to adherence.
Session 3 (50 minutes)	• Goal setting<br>• Review of the sleep diary<br>• Introduction to titration: participants are shown the calculations involved in titrating their sleep schedule based on the previous weeks' sleep diary outcomes. This involves calculating the average nightly total sleep time over the previous week and determining whether sleep efficiency meets a threshold for titrating time in bed up or down, or no change.<br>• Introduction to behavioural stimulus control work: this addresses any conditioned arousal that has developed in bed or bedroom environment during the development and maintenance of one's insomnia. The goal is to eliminate all negative conditioned stimuli that exist between a participant's bed and being awake therefore only using the bedroom for sleeping or sex and avoiding sleeping

	elsewhere (including napping). It also involves reconditioning a positive association between bedroom/bed and sleep.<br>• Adapting stimulus control for individuals with severe stiffness and pain.<br>• Introduction to cognitive work: this involves targeting the racing minds and helping to manage worry before a participant attempts to sleep. It consists of using tools such as a cognitive control diary intended to combat night-time worries that focus on the next day and also cognitive distraction techniques that aim to fill the mind in order to prevent negative, sleep-related, or other related thoughts when falling asleep.
Session 4<br>(50 minutes)	• Goal setting<br>• Review of the sleep diary<br>• Sleep schedule titration<br>• Identifying and assessing dysfunctional beliefs about sleep using motivational interviewing. Motivation interviewing involves moving away from a state of indecision or uncertainty towards finding the motivation to make a positive decision and accomplish established goals.
Session 5<br>(50 minutes)	• Goal setting<br>• Review of the sleep diary<br>• Sleep schedule titration<br>• Review of the cognitive control techniques<br>• Introduction to relaxation techniques: several types of relaxation techniques are discussed in this session with signposting to free resources to help a participant practice these at home.
Session 6<br>(50 minutes)	• Goal setting<br>• Review of the sleep diary<br>• Sleep schedule titration<br>• Review of the relaxation techniques<br>• Review of all sessions covered and focus on relapse prevention. Here the participant and therapist discuss an action plan and the next steps to maintain gains in sleep quality, the continuation of the sleep diary and the next steps of the study are also discussed.
Session 7<br>(optional)	• Unscripted- review of how participant’s sleep scheduling and sleep quality looks like one-month post-intervention.
