# Supplementary material for: Telehealth delivery of adapted CBT-I for insomnia in chronic pain patients: a single arm feasibility study
Source: Front Psychol. 2024 Jan 11;14:1266368. doi: 10.3389/fpsyg.2023.1266368 (PMC10808483; doi:10.3389/fpsyg.2023.1266368)
Supplement: Supplementary file 2 [file Data_Sheet_2.PDF]

Supplementary File A.2 Participant sleep profiles at baseline, post-intervention, and follow-up

Participant	Insomnia type at baseline	Baseline ISI	Post-intervention ISI	Follow-up ISI	ISI Change (baseline-post)	ISI Change (baseline-follow-up)	Baseline SOL (minutes)	Post-intervention SOL (minutes)	Follow-up SOL (minutes)	SOL Change (baseline-post)	SOL Change (baseline-follow-up)	Baseline WASO (minutes)	Post-intervention WASO (minutes)
P1	Onset and maintenance insomnia	26	6	13	-20	-13	100	13.57	17.14	-86.43	-82.86	52.4	17.71
P2	Onset and maintenance insomnia	23	9	9	-14	-14	62.14	57.86	17.86	-4.28	-44.28	62.14	48.57
P3	Onset and maintenance insomnia	16	3	1	-13	-15	51.8	11.29	12.43	-40.51	-39.37	28.8	8.29
P4	Onset and maintenance insomnia	13	7	5	-6	-8	13.57	25	24	11.43	10.43	24.29	29.29
P5	Onset and maintenance insomnia	25	4	9	-21	-16	50.71	12.14	13.57	-38.57	-37.14	132.86	11.43
P6	Onset and maintenance insomnia	14	2	2	-12	-12	30	10.71	17.86	-19.29	-12.14	36	7.43
P7	Onset and maintenance insomnia	13	5	11	-8	-2	22.86	15	10.71	-7.86	-12.15	40.76	11.43
P8	Onset insomnia	20	7	5	-13	-15	68.57	5	5	-63.57	-63.57	22.86	5
P9	Early morning awakenings	12	3	4	-9	-8	8.57	8.57	8.57	0	0	5.71	14

Follow-up WASO (minutes)	WASO Change (baseline-post)	WASO Change (baseline-follow-up)	Baseline TST (minutes)	Post-intervention TST (minutes)	Follow-up TST (minutes)	TST Change (baseline-post)	TST Change (baseline-follow-up)	Baseline SE (%)	Post-intervention SE (%)	Follow-up SE (%)	SE Change (baseline-post)	SE Change (baseline-follow-up)
60	-34.69	7.6	256.8	384	331.2	127.2	74.4	41.02	86.81	72.91	45.79	31.89
26.43	-13.57	-35.71	512.4	370.2	468	-142.2	-44.4	80.96	73	85.23	-7.96	4.27
9.57	-20.51	-19.23	256.2	424.2	411.6	168	155.4	70.57	93.21	91.04	22.64	20.47
42.86	5	18.57	346.2	357	357	10.8	10.8	88.61	85.28	77.31	-3.33	-11.3
13.57	-121.43	-119.29	475.8	469.8	467.4	-6	-8.4	70.93	94.18	93.98	23.25	23.05
20	-28.57	-16	451.8	432.6	463.2	-19.2	11.4	86.39	95.11	91.91	8.72	5.52
14.29	-29.33	-26.47	413.3	466.2	491.4	52.9	78.1	81.85	93.89	94.98	12.04	13.13
5	-17.86	-17.86	369	459	507	90	138	71.37	95.89	97.39	24.52	26.02
5	8.29	-0.71	381.6	396.4	357.6	14.8	-24	91.73	90.99	85.57	-0.74	-6.16

ISI= Insomnia Severity Index, SOL= Sleep Onset Latency, WASO, Wake After Sleep Onset, TST= Total Sleep Time, SE= Sleep Efficiency
